# In Vitro Priming Recapitulates In Vivo HIV-1 Specific T Cell Responses, Revealing Rapid Loss of Virus Reactive CD4^+^ T Cells in Acute HIV-1 Infection

**DOI:** 10.1371/journal.pone.0004256

**Published:** 2009-01-23

**Authors:** Rachel Lubong Sabado, Daniel G. Kavanagh, Daniel E. Kaufmann, Karlhans Fru, Ethan Babcock, Eric Rosenberg, Bruce Walker, Jeffrey Lifson, Nina Bhardwaj, Marie Larsson

**Affiliations:** 1 New York University School of Medicine, New York, New York, United States of America; 2 Partners AIDS Research Center (PARC), Massachusetts General Hospital, Harvard Medical School, Charlestown, Massachusetts, United States of America; 3 SAIC Fredrick, Inc., National Cancer Institute, Fredrick, Frederick, Maryland, United States of America; 4 Molecular Virology, Department of Clinical and Experimental Medicine, Linköping University, Linköping, Sweden; New York University School of Medicine, United States of America

## Abstract

**Background:**

The requirements for priming of HIV-specific T cell responses initially seen in infected individuals remain to be defined. Activation of T cell responses in lymph nodes requires cell-cell contact between T cells and DCs, which can give concurrent activation of T cells and HIV transmission.

**Methodology:**

The study aim was to establish whether DCs pulsed with HIV-1 could prime HIV-specific T cell responses and to characterize these responses. Both infectious and aldrithiol-2 inactivated noninfectious HIV-1 were compared to establish efficiencies in priming and the type of responses elicited.

**Findings:**

Our findings show that both infectious and inactivated HIV-1 pulsed DCs can prime HIV-specific responses from naïve T cells. Responses included several CD4^+^ and CD8^+^ T cell epitopes shown to be recognized in vivo by acutely and chronically infected individuals and some CD4^+^ T cell epitopes not identified previously. Follow up studies of acute and recent HIV infected samples revealed that these latter epitopes are among the earliest recognized in vivo, but the responses are lost rapidly, presumably through activation-induced general CD4^+^ T cell depletion which renders the newly activated HIV-specific CD4^+^ T cells prime targets for elimination.

**Conclusion:**

Our studies highlight the ability of DCs to efficiently prime naïve T cells and induce a broad repertoire of HIV-specific responses and also provide valuable insights to the pathogenesis of HIV-1 infection in vivo.

## Introduction

HIV-specific cellular immune responses play a central role in controlling HIV-1 replication and in delaying disease progression in infected individuals. The importance of CD4^+^ and CD8^+^ T cell responses is highlighted in longterm nonprogressors (LTNPs), whose ability to control infection is correlated with the presence of strong and broadly directed HIV-specific T cell responses [1], [2], [3]. The presence of activated and proliferating CD4^+^ CCR5^+^ gag specific T cells, expressing perforin and granzyme B, in early primary infection supports a potential role for them in helping to control viral replication [4]. However, these T cells disappear as the infection progresses. Similar virus-specific perforin expressing CD4^+^ T cells exist in rhesus macaques infected with attenuated strains of SIV which protected them from virulent wild type virus challenge [5]. These studies highlight the important role T cells, more specifically CD4^+^ T cells, can play in determining the course of infection.

Dendritic cells (DCs) are the only APC capable of priming naïve T cells in vivo and turning them into long-lasting functional memory T cells. The magnitude and strength of a generated T cell response is based on the quality of the initial priming event, which in turn depends on several factors including trafficking of DCs to lymph node[6] and the quality of the initial DC-T cell interaction[7]. Vaccine studies have demonstrated that it is possible to activate already existing memory cells and prime new T cell responses in vivo [8], [9], [10]. A recent study used an autologous vaccine consisting of DC pulsed ex vivo with AT-2 inactivated HIV-1 (AT-2 HIV-1) provided promising data with induction of increased levels of HIV-specific T cells and decreased viral load in chronically infected subjects[10]. Thus, DCs appear to be central in the generation of protective immunity against HIV-1.

Previous studies examining in vitro HIV-1 T cell priming have used MDDCs pulsed with different HIV-antigenic constructs, such as peptides, proteins, liposome complexed proteins and cDNA [11], [12], [13], [14] to prime naïve T cells. However, the capacity of DCs pulsed with whole virions; the most physiological source of HIV-antigens, to prime T cells in vitro has not been investigated. Here, we examined whether infectious and AT-2 HIV-1, with functional binding and fusion abilities, can serve as efficient sources of antigen for DCs to prime HIV-specific T cells from naïve cells in vitro. The use of AT-2 HIV-1 as a source of antigen may be particularly relevant as the majority of virions found in circulation do not possess culturable infectivity [15], [16], [17], [18]. Even if not infectious, these viruses may serve as an important source of antigens in vivo. Using both infectious and AT-2 HIV-1 also allowed us to compare the nature of responses generated with these antigen sources, findings of great interest for vaccine design.

Our findings show that DCs loaded with AT-2 or infectious HIV-1 primed HIV-specific responses from naïve T cells from uninfected individuals in vitro. Priming with AT-2 HIV-1 yielded slightly more frequent responses. MDDCs pulsed with HIV-1 stimulated significantly more CD4^+^ than CD8^+^ T cell responses. The specificities of the primed T cells consisted of epitopes located in env gp41 and gp120, gag p17 and p24, and pol INT and RT. For the CD4^+^ T cell responses, five out of ten of the in vitro primed responses matched the responses seen ex vivo in HIV-infected individuals at different stages of disease[19]. Furthermore, we identified several novel HIV-1 CD4^+^ T cell responses in env and pol, and we were able to detected 4 out of 5 of these novel responses in acute and recent HIV-infected individuals. The overwhelming CD4^+^ T cell responses seen in our in vitro priming are consistent with the strong and broad CD4^+^ T cell responses we have observed in individuals with acute HIV-infection and which disappeared or were greatly reduced within three months after onset of infection. Although less frequent, CD8^+^ T cell responses were also observed. Of note, all in vitro primed CD8^+^ T cell responses matched the responses seen ex vivo in HIV-infected individuals at different stages of disease [19], [20]. Our study shows that DCs pulsed with HIV-1 can prime the same responses that arise naturally in vivo in HIV-infected individuals and demonstrates that AT-2 HIV-1 is a excellent source of antigens that may have the capacity in a vaccine setting, not only to restimulate existing memory responses but also prime for new T cell responses or re-establish responses lost very early on during infection.

## Results

The aims of this study were to establish whether DCs pulsed with infectious or chemically inactivated HIV-1 could prime HIV-specific T cell responses, examine the quality of primed T cell responses, and compare them with the responses observed in vivo in primary HIV-infection. Infectious and noninfectious (AT-2 HIV-1) virions of HIV-1_MN_, were tested side by side to evaluate priming efficiency and qualitative characteristics of the T cell responses elicited. HIV-1 specific priming was performed using MDDCs and naïve CD45RA^+^ CD62L^+^ bulk T cells from PBMCs from 11 HIV-negative individuals.

### Exposure of mature MDDCs to AT-2 or infectious HIV-1 does not reverse their phenotypic and functional maturation

**Figure 1:**
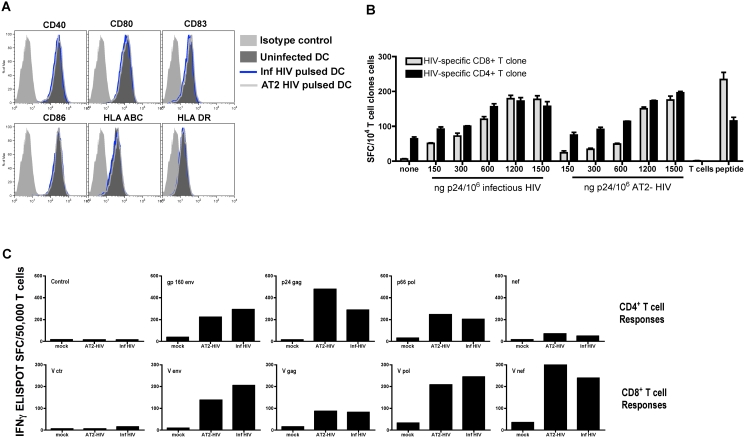
HIV-1 does not affect the phenotypic maturation of MDDCs or their T cell stimulatory ability. (A) Matured MDDCs were unpulsed or pulsed with 1500ng/10^6^ p24 equivalents of AT-2 HIV-1 or infectious HIV-1 for 16–18hrs and washed. The effect of HIV-1 on the mature MDDCs was assessed by staining with isotype control, CD40, CD80, CD83, CD86, HLA ABC, and HLA DR antibodies conjugated to phycoerythrin (PE).B) Mature MDDCs were unpulsed or pulsed with 150ng–1500ng/10^6^ p24 equivalents of AT-2 HIV-1 or infections HIV-1 for 16–18 hrs. The ability stimulate T cells was measured using a HIV-specific CD8^+^ T cell clone recognizing HLA A2.01-restricted SL9 gag p17 and a HIV-specific CD4^+^ T cell clone recognizing HLA DR4-restricted LI15 gag p24. The activation of T cells was measured by IFN- γ Elispot assay. (C) Mature MDDCs from a chronically infected individual were pulsed with 300ng/10^6^ p24 equivalents MV, AT-2 or infectious HIV-1 and cocultured with autologous T cells for 7 days at a DC:T cell ratio of 1:10. At day 7, the different groups of T cells were harvested and restimulated with mature MDDCs infected with vaccinia vector constructs (V ctr, V gag, V pol or V nef) to detect CD8+ T cell expansion or MDDCs pulsed with recombinant proteins (ctr, gp160, p24, p66 or nef) to detect CD4+ T cell expansion. The responding T cells were enumerated by IFN-γ Elispot assay. The data are representative of 4 experiments.

The contact between the virus and DC subpopulations likely represents a fundamental part of HIV-1 pathogenesis and may affect the DCs phenotype and ability to stimulate T cells [21], [22], [23], [24]. To evaluate potential deleterious effects of HIV-1 exposure on the antigen presenting properties of DCs to be used for T cell priming, we assessed the effect of HIV-1 on the viability and expression of costimulatory molecules of matured MDDC after overnight incubation with AT-2 or infectious HIV-1_MN_. At the dosage of virus used in the subsequent experiments, 150ng p24 equivalent/10^5^ cells, we did not observe any negative effect on the viability (data not shown), maturation status (**Figure 1A**) or ability to activate HIV-specific T cell clones **(** **Figure 1B**). Furthermore, the ability of MDDCs pulsed with either infectious or AT-2 HIV-1 to activate and expand CD4^+^ and CD8^+^ HIV-specific T cells from chronic HIV-infected individuals was examined and DCs pulsed with either form of virus expanded broad HIV-1 specific memory T cell responses targeting major HIV antigens gag, pol, env, and nef **(** **Figure 1C** and Larsson et al[25] **)**. Additionally, our previous study established that both forms of virus are processed and presented in a similar manner using, for the most part, the classical pathways for MHC class I and II presentation [26]. These findings establish that the ability of DCs to function as APCs is preserved in this system, both for infectious and AT-2 HIV-1.

### Both infectious and AT-2 inactivated HIV-1 can be used as antigenic sources by DCs to prime HIV-specific T cells from CD45RA+ naïve T cells

**Figure 2:**
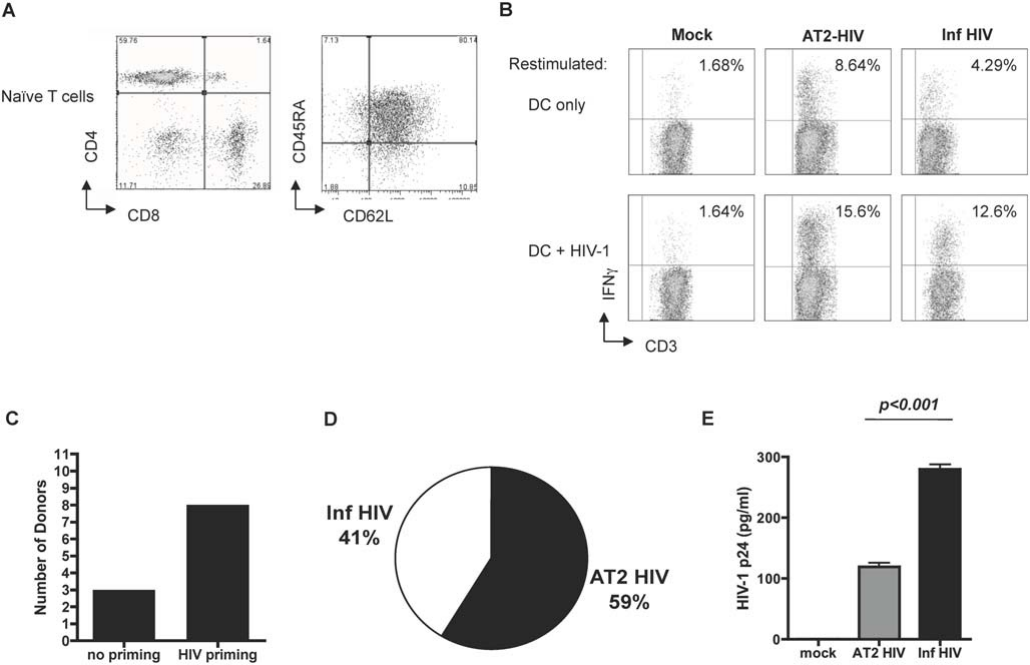
HIV-1 pulsed MDDCs prime HIV-1 specific T cells. (A) Autologous naïve bulk (CD4^+^ and CD8^+^) T cells were negatively isolated from PBMCs by magnetic beads to remove CD14, CD19, CD56 and CD45RO positive cells. (B) The autologous naïve bulk T cells were co-cultured with the mock (no virus), AT-2 or infectious HIV-pulsed DCs. Cultures were restimulated weekly by the addition of DCs pulsed with mock, AT-2 HIV-1 or Inf HIV-1 for 4 weeks. HIV-specific responses were detected by intracellular cytokine staining for IFN-γ after 4 weeks of culture. (C) HIV-1 specific priming was attempted with bulk T cells from 11 HIV naïve donors to establish the efficiency of in vitro MDDC priming. 8 out of 11 donors were successfully primed. (D) The priming efficiency of MDDCs pulsed with AT-2 HIV-1 vs. infectious HIV-1 of the 8 donors that was successfully primed were compared. (E) Priming cultures were analyzed for the presence of viral replication. Supernatants were collected from the cultures after the 3^rd^ restimulation on day 21 and virus replication was measured using HIV p24 ELISA kit. Mock, AT2-HIV, and Inf HIV priming cultures were compared.

The ability of DCs to prime HIV-specific responses in vitro has previously been established by several studies using various antigen preparations, e.g. peptides, proteins, liposome complexed proteins and cDNA[11], [12], [13], [14] but not whole virions, the most physiologically relevant source of HIV-antigens. We tested whether we could prime HIV-specific T cells in vitro using an autologous system consisting of mature DC, pulsed with AT-2 or infectious HIV-1, and naïve CD45RA^+^ CD62L^+^ bulk T cells **(** **Figure 2A** **)** from uninfected individuals. HIV-priming was usually achieved after the third or fourth restimulation, as measured by intracellular IFN-γ staining after antigenic rechallenge, **(** **Figure 2B** **)** and considered positive when the level of responding cells, i.e. IFN-γ producing T cells, was >1%–5% above the non-specific IFN-γ production. Priming was achieved in 8 out of the 11 donors examined **(** **Figure 2C** **),** and reasons for the failure to induce priming in all donors are unknown, but may include the death of newly activated T cells, the HLA differences among donors, or even T cell impairing effects exerted by the virus on the immune cells from some individuals. Cultures using unpulsed DCs served as controls for the specificity of the HIV-priming assays and no significant HIV-specific responses were observed in these mock-primed cultures **(** **Figure 2B** **)**. Of note, we observed a background production of cytokines when the HIV-1 primed T cells were tested with unpulsed DCs and this is attributed to T cells still active from the weekly restimulation with HIV-1 pulsed DC as the culture with mock DC did not have this production.

We have shown equivalent efficiency of MDDCs to process and present antigens derived from AT-2 HIV-1 or infectious HIV-1 as measured by activating HIV-specific memory T cells from chronic HIV infected individuals[27] **(** **Figure 1C** **)**. However, as the source of antigens for the in vitro priming of naïve T cells by MDDCs, AT-2 HIV-1 was slightly more efficient, 59% of all primed responses was achieved using AT-2 HIV-1 compared to 41% by its infectious counterpart **(** **Figure 2D** **)**. This outcome may be attributed to negative immune regulatory effects exerted by infectious HIV-1 on newly activated T cells and/or effects on the DCs in these long term cultures [24], [28], [29]. Of note, we observed low levels of replication in the priming cultures with infectious HIV compared to priming cultures with AT2-HIV **(** **Figure 2E** **)**. Our study demonstrates for the first time that HIV-1 pulsed DCs can prime HIV-specific T cells from naïve donors and that AT-2 inactivated HIV-1 was slightly more efficient than infectious HIV-1 as an antigenic source.

### DCs pulsed with infectious or AT-2 HIV-1 primed polyfunctional T cell responses that target a broad repertoire of HIV-antigens

**Figure 3:**
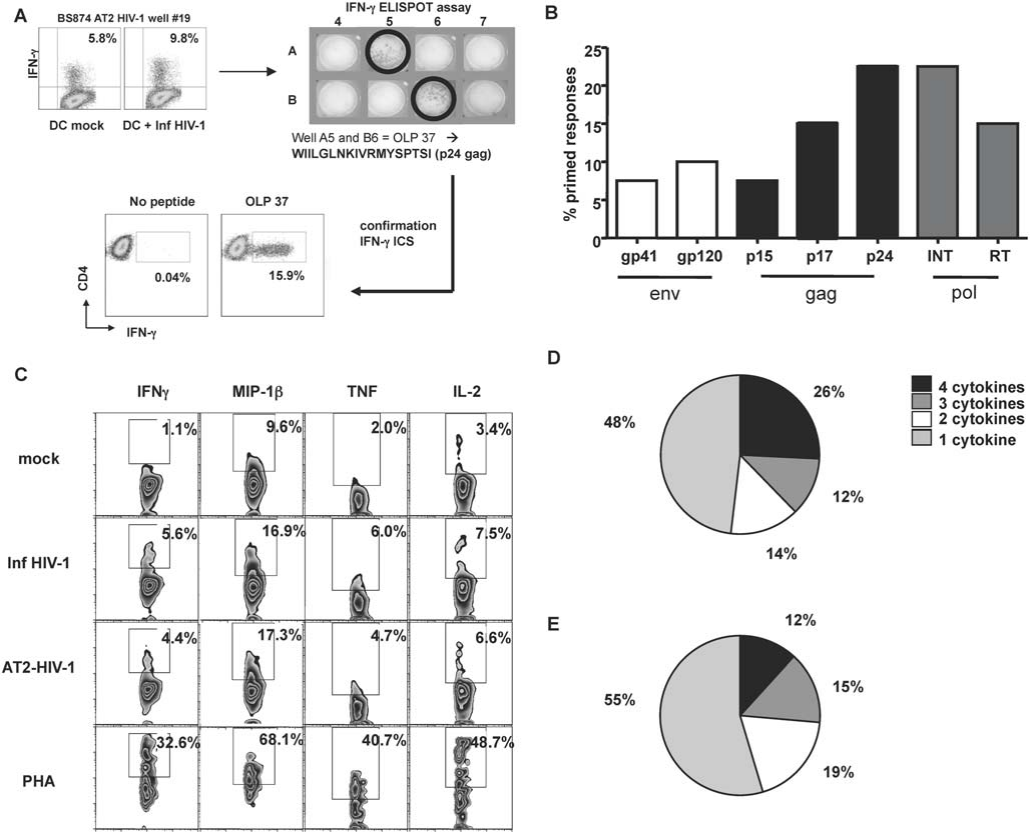
HIV-specific T cells are polyfunctional and recognized a broad repertoire of antigens. (A) Mapping of epitopes was done using pools of overlapping peptides. The peptides spanned the entire HIV-1 genome and were based on the consensus B sequence (http://www.hiv.lanl.gov/content/immunology/index.html). The peptide pool matrices and the HIV-1 primed T cell cultures were added to IFN-γ Elispot assays as described previously [19]. The assays were developed after overnight culture and evaluated. Reconfirmations of all positive wells were done the following day with the single peptide by flow cytometry to detect intracellular IFNγ production. (B) The reconfirmed HIV-1 specific T cell responses from all donors tested were analyzed to determine which HIV-1 antigens the primed T cells recognized and the percentile they constituted of total primed responses from those screened. (C) The effector functions of the HIV-1 specific polyclonal T cells were examined by stimulation with MDDCs pulsed with AT-2 HIV-1 or infectious HIV-1 and the intracellular production of the effector cytokines/chemokines; IFN-γ, TNF-α, IL-2 and MIP-1β, was measured by flow cytometry. The polyfunctionality of the T cell responses were analyzed as the ability to produce the combination of one, two, three or all four of the cytokines/chemokines tested by the primed CD4^+^ (D) and CD8^+^ (E) T cells in response to HIV.

The requirement for initiation and maintenance of broad and strong T cell responses capable of controlling viral load is clear when examining the HIV-infected individuals that do succeed to control the virus for many years [1]. To establish the antigenic repertoire of the primed T cells, the HIV specific polyclonal T cell populations were evaluated by IFN-γ Elispot using a set of overlapping peptides spanning the complete HIV Clade B consensus sequence **(** **Figure 3A** **).** We found broad responses targeting epitopes located in gag MA (15%), CA (8%), pol INT (22%), RT (15%), env gp120 (10%) and gp41 (8%) **(** **Figure 3 B** **,** **Table 1** **)**. In most cases, primed T cells from various donors recognized as few as two epitopes or as many as nine. For instance, the specific responses we detected in donor MP880 were located in env gp41, env gp120, gag MA (p17), gag CA (p24) and pol INT, thus demonstrating that within one donor a broad repertoire of HIV-specific T cell responses can be primed using MDDCs.

**Table 1:** Primed CD4 and CD8 T cell responses

Polyclonal T cell Lines	Virus	Protein	OLP Sequence
BS874 AT2 #19	MN AT2	gag/p24	WIILGLNKIVRMYSPTSI
BS874 AT2 #21	MN AT2	gag/p17	TGSEELRSLYNTVATL
BS874 AT2 #23	MN AT2	gag/p24	WIILGLNKIVRMYSPTSI
BS874 AT2 #24	MN AT2	gag/p24	WIILGLNKIVRMYSPTSI
			YVDRFYKTLRAEQASQEV
BS874 AT2 #25	MN AT2	gag/p24	WIILGLNKIVRMYSPTSI
BS874 LHIV #14	MN LHIV	gag/p24	WIILGLNKIVRMYSPTSI
		pol/RT	MTKILEPFRRKQNPDIVIY
BS874 LHIV #17	MN LHIV	gag/p24	WIILGLNKIVRMYSPTSI
		pol/RT	MTKILEPFRRKQNPDIVIY
BS874 LHIV #35	MN LHIV	gag/p15	RNQRKIVKCFNCGKEGHT
MP980 LHIV #9	MNLHIV	gag/p17	TGSEELRSLYNTVATL
MP980 LHIV #16	MN LHIV	pol/INT	ELKKIIGQVRDQAEHLK
MP980 AT2 #7	MN AT2	env/gp41	LELDKWASLWNFDITN
		gag/p17	KHIVWASRELERFAV
MP980 AT2 #9	MN AT2	gag/p24	WIILGLNKIVRMYSPTSI
MP980 AT2 #36	MN AT2	env/gp41	AVLSIVNRVRQGYSPLSE
		env/gp120	RPVVSTQLLLNGLSLA
MP980 AT2 #48	MN AT2	gag/p17	SGGELDRWEKIRLRPGGK
DR682 AT2 #1	MN AT2	pol/INT	TKELQKQITKIQNFRVYY
		gag/p24	IVQNLQGQMVHQAISPR
DR682 AT2 #3	MN AT2	gag/p17	IVQNLQGQMVHQAISPR
		pol/INT	AYFLLKLAGRWPVKTIH
			LKTAVQMAVFIHNFKRK
DR682 AT2 #5	MN AT2	gag/p24	WIILGLNKIVRMYSPTSI
DR682 AT2 #7	MN AT2	env/gp41	LELDKWASLWNFDITN
JP651 LHIV #13	MN LHIV	gag/p24	IVQNLQGQMVHQAISPR
		gag/p15	HIAKNCRAPRKKGCWK
EW836 LHIV #5	MN LHIV	pol/RT	YELHPDKWTVQPIVLPEK
EW836 AT2 #4	MN AT2	gag/p17	LEKIEEEQNKSKKKAQQA
JN521 AT2 #1	MN AT2	gag/p15	GKIWPSHKGRPGNFLQSR
		gag/p17	MGARASVLSGGELDRWEK

We examined if the HIV-1 specific polyclonal cell lines, selected based on the production of IFN-γ, had the ability to produce other effector cytokines such as IL-2, TNF-α or MIP-1β when stimulated with MDDCs exposed to HIV-1 **(** **Figure 3C** **)**. The HIV-specific CD4^+^ T cells **(** **Figure 3D** **)** and CD8^+^ T cells **(** **Figure 3E** **)** in the polyclonal population were found to produce one to four cytokines upon stimulation. Taken together, these findings demonstrate that the primed CD4^+^ and CD8^+^ T cells target a broad repertoire of HIV-1 antigens and that they are polyfunctional in their ability to secrete multiple cytokines upon stimulation.

The responses most frequently detected in HIV-1 infected individuals, independent of the stage of the disease, are gag p24- and nef-specific T cells[30]. Besides yielding the highest frequency of HIV-specific T cells, these two proteins also contain the highest epitope density [20], [30]. Our in vitro priming induced a variety of p24-specific responses and they constituted 22% of all HIV-1 specific responses primed. However, in contrast to Addo et al [30] no nef specific responses were confirmed in our assays. The reason for this discrepancy between the in vivo and the in vitro responses is unclear. However, we can rule out the possibility that the DCs are unable to process and present the nef protein as we have previously shown that DCs can activate nef specific memory T cells when pulsed with HIV-1 **(** **Figure 1C** **)** [25] **.** Alternatively, the lack of nef responses may be attributed to lack of viral replication within the priming cultures. Relatively little nef is found in virions and in the absence of robust productive infection, there may be insufficient antigen available for priming, particularly compared to more abundant proteins like gag, pol, and env.

### The specificity of the in vitro primed T cell responses reflect those seen in infected individuals primed in vivo

**Figure 4:**
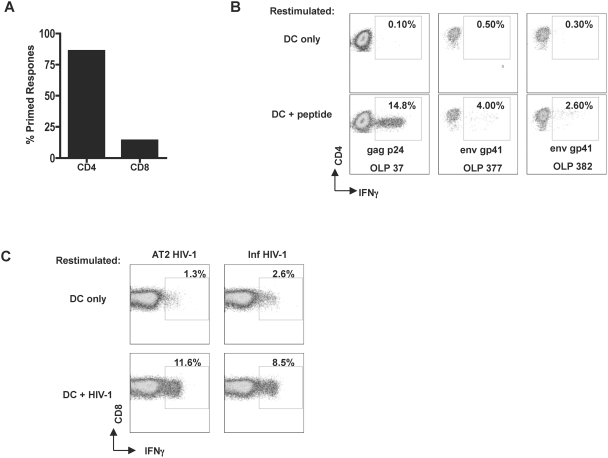
The majority of the responses primed by MDDCs are HIV-1 specific CD4+ T cell responses. (A) The HIV-1 specific T cell responses from all donors tested were characterized for expression of CD4 or CD8 and plotted as the percentile of total confirmed primed responses. (B). Single peptide confirmation of HIV-1 specific T cells were done with the specific peptide by flow cytometry to detect intracellular IFNγ production. The dot plots shows HIV-1 specific CD4^+^ T cell responses for gag OLP 37, gp41 OLP 377 and gp41 OLP 382. (C) HIV-1 specific priming of CD8^+^ T cells. CD8^+^ T cells were obtained by flow cytometry sorting. After the 4th restimulation, HIV-specific responses were detected by intracellular cytokine staining for IFN-γ.

Several studies have analyzed the specificity of HIV-1 memory T cell responses existing in infected subjects by ex vivo assays [19], [31], [32] and the CD8^+^ T cells are so far the most extensively studied [32], [33]. Less is known about HIV-specific CD4^+^ T cells as these are specifically targeted by the virus as the infection progresses [34], [35]. The majority (86%) of the confirmed primed responses were CD4^+^ T cells **(** **Figure 4A** **).** The basis for this CD4^+^ skewing could be attributed to several factors including use of whole virions as the source of antigens, and the in vitro system used for delivery of the virus. In addition, CD4^+^ T cell responses are the first to arise in vitro and may thus overwhelm the CD8^+^ T cells. However, we can rule out the possibility that our in vitro system merely selects for the development of HIV-specific CD4^+^ T cells. Though they made up a minority of the responses observed, we did see unequivocal development of HIV-specific CD8^+^ T cells. Furthermore, when we depleted CD4^+^ T cells from the co-culture system, we saw enhancement of priming of CD8^+^ T cells **(** **Figure 4C** **)**. Therefore, it is possible to prime HIV-specific CD8^+^ T cells using our in vitro system.

In our in vitro priming, five out of ten CD4^+^ T cell responses matched the responses previously documented ex vivo with cells from HIV-1 infected subjects at different stages of disease and a number of these HIV-1 specific CD4^+^ T cell responses are frequently detected **(** **Table 2** **:** **Figure 4B** **)** [19]. The primed p24 epitopes WIILGLNKIVRMYSPTSI and YVDRFYKTLRAEQASQEV are frequently observed in HIV-infected subjects[19], [30] and p24 contains several CD4^+^ T cell epitopes[19]. For instance, the WIILGLNKIVRMYSPTSI p24_133-150_ response was frequently seen in the majority of the donors we tested and several T cell clones responding to epitope/s located within p24 have been established (data not shown). p24_133-150_ is contained within the immunodominant region of gag and also contains several known CD8-epitopes including GLNKIVRMY and IILGLNKIVR. Moreover it contains multiple CD4+ epitopes that are promiscuous and can be loaded on to several different HLA DR haplotypes (e.g. ILGLNKIVRMY recognized in DRB1*0101, DRB1*1302, DRB1*1501) a factor that may contribute to its frequent recognition. In addition, the peptide YVDRFYKTLRAEQASQEV has been reported to be the peptide most recognized by CD4^+^ T cells in all categories of subjects investigated[19].

**Table 2:** Correlation of primed T cell responses with in vivo data

T cell Type	HIV Protein	HXB2 location	OLP sequence	Epitopes (HLA-restriction)	Stage of disease	References
CD4	Gag p17	9–26	SGGELDRWEKIRLRPGGK	Human	Mostly in acute Lower responses in chronic High responses in untreated	▪*Kaufmann et al J Virology 2004*
CD4	Gag p17	32–46	KHIVWASRELERFAV	Human		▪*Wilson et al J Virology 2001*
CD4	Gag p17	70–86	TGSEELRSLYNTVATLY	Human		▪Los Alamos HIV Immunology Database
CD4	Gag p24	133–150	WILLGLNKIVRMYSPTSI*	DRB1*1501, DRB1*1302, DRB1*0401, DRB5*0101, DRB1*1101, DRB1*0701, DRB1*0405, DRB1*0101		
CD4	Gag p24	164–181	YVDRFYKTLRAEQASQEV	DRB1*1501, DRB1*1302, DRB1*0401, DRB5*0101, DRB1*1101, DRB1*0701, DRB1*0405, DRB1*0101		
CD4	Pol INT	210–277	TEKLQKQITKIQNFRVYY	Human, DR supermotif		
CD8	Gag p17	9–26	SGGELDRWEKIRLRPGGK	A* 0301, A3, B*4002, B7, B27, B40, B63	Found in all stages of disease	▪*Addo et al J Virology 2001*
						▪*Frahm et al J Virology 2005*
CD8	Gag p17	70–85	TGSEELRSLYNTVATL	A*0101, A1, A2, A*0201, A*0202, A*0205, A*0214, A24, B*4006, B*0801, B8		▪Los Alamos HIV Immunology Database

Studies on the antigenic specificity of CD8+ T cell responses in HIV-1 infected subjects, especially in chronic infection, are more comprehensive [31], [32]. Responses to three in vivo HIV-1 specific CD8^+^ T cell epitopes described in HIV-1 infected patients were primed in our in vitro system and all were gag specific. They are located within gag p15 and p17, corresponding to TGSEELRSLYNTVATL p17_70-85_ and SGGELDRWEKIRLRPGGK p17_9-26_ and RNQRKIVKCFNCGKEGHT p15_21-38_ **(** **Table 2** **).** These peptides contain at least five epitopes that have been identified ex vivo in HIV-1 infected subjects at different stages of disease [19], [31], [36] (Los Alamos NL). CD8^+^ T cells specific for the TGSEELRSLYNTVATL and SGGELDRWEKIRLRPGGK p17 epitopes have also previously been primed for in vitro using DCs pulsed with liposome complexed HIV-1 protein[11] and DC pulsed with peptide[12]. The CD8^+^ T cell responses primed with peptide pulsed DCs recognized the epitope KIRLRPGGK within the p17 SGGELDRWEKIRLRPGGK sequence and this is a HLA*A3-restricted epitope[12]. Our response was seen in a HLA* A3-positive subject and thus could be directed to the same CD8^+^ T cell epitope. Alternatively, it could be a novel epitope response contained within the same region. Thus comparison of our data with ex vivo data obtained from HIV-infected subjects demonstrates that the epitopes seen in our in vitro priming correlates with T cell responses that develop in vivo.

### Strong and broad HIV-specific CD4 T cell responses can be identified at time of acute HIV-infection but quickly decline irrespective of treatment status

**Figure 5:**
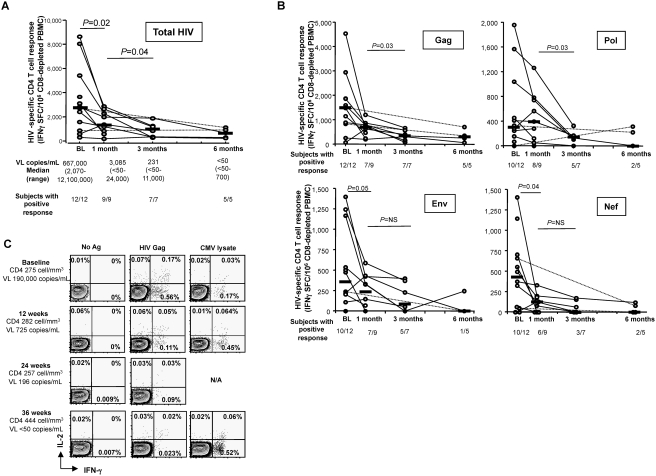
Strong and broad HIV-specific CD4 T cell responses exist in acute infection which quickly diminish. (A and B) Fresh CD8-depleted PBMCs from 12 adult individuals with documented primary HIV infection (PHI) were screened for HIV Gag-specific CD4 responses by IFNγ Elispot using a pool of overlapping peptides spanning all HIV proteins and corresponding to Clade B consensus sequence 2001. Subjects were assessed at baseline (BL) before institution of any antiviral therapy and 1 month, 3 months and 6 months after BL. Data represent total HIV-specific CD4 T cells responses (A) as well as responses to individual gene products (B). Grey circles: subjects treated with HAART; white circles: individuals who remained without therapy. Horizontal black bars: median values. (C) This decline can also be demonstrated by intracellular cytokine staining and affects both IFNγ- and IL-2-secreting CD4+ T cells. Fresh PBMCs from an adult individual with acute HIV infection were stimulated with medium alone, the same HIV Gag as above or a CMV lysate, in the presence of anti-CD28 and anti-CD49 stimulating antibodies and submitted to intracellular cytokine staining after six hour incubation. Similar experiments were performed 12, 24 and 36 weeks after BL. These results are representative of three separate subjects. Numbers in quadrants: percentage of cytokine-producing CD4^+^ T cells.

The predominantly CD4^+^ T cell responses and the observation of in vitro primed specificities that had not been previously reported from studies of infected patients prompted us to re-examine in vivo responses more closely, in particular in acute HIV-infected subjects. In order to assess the repertoire of HIV-specific CD4^+^ T cell responses found in acute HIV infection (AHI), we used fresh CD8-depleted PBMCs from 12 subjects with documented AHI and screened them for HIV-specific CD4^+^ T cell responses. Peptides were divided in peptide pools corresponding to the different HIV proteins before being used in the Elispot assays and responses were analyzed per protein and as total response obtained by summing up the responses to the individual HIV proteins. Furthermore, we monitored changes in CD4^+^ T cell responses over time. At the first available time point for immunological studies and before initiation of HAART (baseline, BL), all investigated subjects showed a positive, and in most cases robust, HIV-specific CD4^+^ T cell response (median total response 2,780 SFC/10^6^ CD8-depleted PBMCs; range 315-8646) **(** **Figure 5A**). At one month after BL, 8 out of 9 evaluated subjects showed a decrease in magnitude of the HIV-specific CD4^+^ T cell responses compared to baseline (p = 0.02; Wilcoxon matched pairs test)Further decline was observed 3 months after BL compared to the one month time point in 6 of 7 individuals studied longitudinally (p = 0.04; Wilcoxon matched pairs test). The HIV-specific CD4^+^ T cell responses waned in both subjects treated with HAART (grey circles) and in individuals who remained without therapy (white circles) and occurred while most subjects were still viremic, showing that the reduction in HIV-specific CD4^+^ T cell responses occurred in spite of a continuous exposure to the antigen.

In order to investigate the responses to the different proteins, we next analyzed the evolution of the CD4^+^ T cell responses to the Gag, Pol, Env and Nef proteins (**Figure 5B** **).** The results demonstrate that HIV-specific CD4^+^ T cell responses at the time of acute infection target various proteins and that the overall responses declined over time. Gag-specific responses remained detectable six months after AHI whereas most subjects had lost their Pol-, Nef-, and Env-specific responses at this time point. Of note, the magnitude of Env-specific CD4^+^ T cell responses detectable during AHI is remarkable compared to their rarity in chronic infection [37]. We used intracellular cytokine staining for IFN-γ and IL-2 to confirm this decline in HIV-specific CD4^+^ T cell responses after AHI with another experimental approach **(** **Figure 5C** **).** The strong IFNγ- and IL-2 HIV-Gag-specific CD4^+^ T cell response present at baseline before institution of therapy declined over time, contrasting with the stability of the CMV- specific CD4^+^ T cell response, demonstrating that this phenomenon is HIV-specific. Thus, robust and broad HIV-specific CD4^+^ T cell responses can be detected during AHI however, they quickly decline. In particular, Env-specific CD4^+^ T cell responses, which are not commonly detected in chronic HIV-infected subjects, are readily detected during AHI. However, these responses disappear very quickly. Therefore, the CD4^+^ T cell responses we saw in our priming could be a reflection of what is happening during acute infection whereby broad HIV-1 specific CD4^+^ T cell responses are activated very early on, even before the development of HIV-specific CD8^+^ T cells and decline as activated CD4^+^ T cells are directly or indirectly destroyed by the virus.

### Novel epitopes primed for in vitro correlated with CD4 responses that arise and are present during acute and recent HIV-1 infection

**Figure 6:**
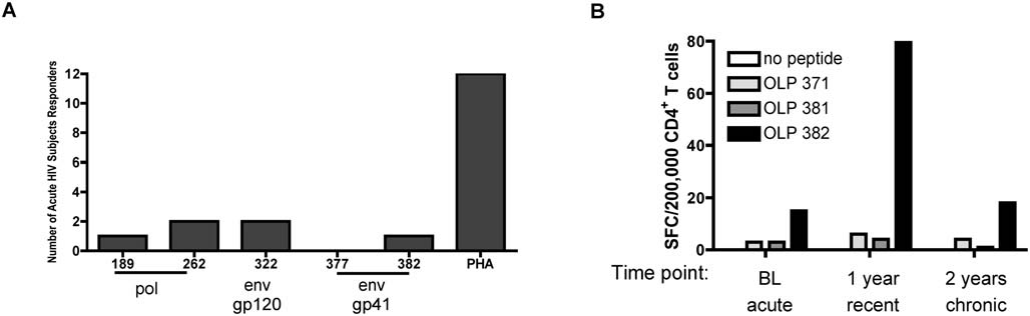
In vitro primed novel HIV epitopes correlates with CD4^+^ T cell responses seen during primary infection. (A) Summary of responses to the novel epitopes detected in 12 acute and recent HIV-infected subject PBMCs. PBMCs from acute and recent HIV infected individuals were thawed and tested directly ex vivo for reactivity to OLPs 11, 189, 262, 322, 377, and 382 using intracellular staining for IFN-γ, TNF-α, and IL-2 after six hour incubation. Reactivity against the peptide was determined when the percentage of cytokine-secreting cells were at least twice the percentage of cytokine-secreting cells in the unstimulated wells. (B) HIV-infected PBMCs from one donor evaluated at 3 different time points after infection (sample one: 2 weeks after onset of retroviral syndrome: acute infection, sample two: 1 year infected: recent infection, and sample three: 2 years infected: chronic infection) were thawed and cocultured with peptide pools OLP 371-381 or OLP 382-392 for 14 days. The peptide activated PBMCs were then assessed for reactivity to each of the individual peptides in the pools used.

Our findings that the CD4^+^ T cell responses decline rapidly in acute HIV-infected subjects could explain why several of the CD4^+^ T cell responses we succeeded to prime for in vitro have not previously been described in the literature. The novel HIV-1 T cell responses we found in env and pol are contained within peptides ELKKIIGQVRDQAEHLK in pol IN_157-173_, MTKILEPFRKQNPDIVIY in pol RT_163-181_, AVLSIVNRVRQGYSPLSF env gp41_700-717_, LELDKWASLWNWFNITNW in env gp41_601-678,_ and RPVVSTQLLLNGSLA in env gp120_252-266._ The ELKKIIGQVRDQAEHLK in pol IN, MTKILEPFRKQNPDIVIY in pol RT and AVLSIVNRVRQGYSPLSF env gp41 have so far been found in only 1 out of 36 chronic HIV infected subjects tested **(data not shown)**. In addition, screening of acute and recent HIV-infected subjects revealed that CD4^+^ responses to 3 of 5 novel epitopes within pol and env are readily detected. We have been able to confirm CD4^+^ T cell responses to epitopes contained within peptides ELKKIIGQVRDQAEHLK in pol IN_157-173_, RPVVSTQLLLNGSLA in env gp120_252-266_, and AVLSIVNRVRQGYSPLSF in env gp41_700-717_ **(** **Figure 6A** **)** when we screened subjects directly ex vivo that were HIV-1 infected less than 6 months. We were unable to confirm more of our novel epitopes in more HIV-infected subjects due to the fragile nature of CD4^+^ T cells at this early time point of infection and difficulty of obtaining enough CD4^+^ T cells from acute HIV-infected subjects. With one acute HIV-infected subject from whom we received follow-up samples, we detected CD4^+^ T cell responses to epitopes contained within peptide AVLSIVNRVRQGYSPLSF in env gp41_700-717_ at the initial time point however, screening of PBMCs from subsequent time points revealed the rise and decline of responses targeting epitopes within this particular peptide **(** **Figure 6B** **)**. Nonetheless, we provide evidence that novel epitopes seen in our in vitro priming may represent T cell responses seen in AHI that disappear in the infected individuals so early on that they are gone in most individual at time of their HIV-1 diagnosis.

Taken together, our data establishes that MDDCs presenting either infectious or AT-2 HIV-1 derived antigens can prime polyfunctional CD4^+^ and CD8^+^ HIV specific T cells that target a broad repertoire of HIV-antigens. Furthermore, we show that our primed HIV-specific T cells correlate to T cells that arise naturally in vivo in HIV-1 infected individuals, more specifically during AHI and is lost early on. Though noninfectious HIV-1 had slightly higher efficiency in priming, there were no significant differences in terms of the type of responses elicited thus demonstrating that defective viruses, can serve as a source of antigen in vivo.

## Discussion

The cellular immune response developed against HIV-1 is multifaceted. DCs in the lymph nodes probably contribute both to the initial strong T cell responses and at the same time to the defects seen in CD4^+^ T cells during AHI. One mechanism explaining the efficiency of viral spread is the formation of the infectious synapse whereby DC harboring HIV-1 interacts with T cells [28], [38]. In this setting, the activated HIV-specific CD4^+^ T cell, expressing CCR5 and high levels of CD38, are prime targets for infection in vivo during primary HIV-infection[39], which explains the preferential infection of HIV-1 specific CD4^+^ T cells[35].

The HIV-1 specific CD4^+^ T cells rapidly decline two and three weeks following the onset of the acute viral illness. This may depend on that 80–90% of these cells lack CD127 during primary HIV-infection, consistent with a predetermined apoptotic fate [39]. In spite of the rapid loss initially, HIV-1 specific CD4^+^ T cells do persist in individuals at all stages of HIV-infection and comprise from 0.02%–2% of the peripheral blood CD4^+^ T cells. The majority of the CD4^+^ T cells that remain in individuals with persistent viremia have defective proliferation ability due to impaired development of IL-2 producing T_CM_ cells [40], PD-1 and CTLA-4 expression [29], [41].

While the exact proportions of infectious and non-infectious virus circulating in vivo remain the subject of some controversy. The role of these noninfectious virions as antigen for priming immune responses has not been explored. Therapeutic vaccination of SIV-infected rhesus monkeys [42], [43] and chronically HIV-infected subjects[10] with autologous DCs pulsed with AT2-inactivated virus was associated with reduced viral loads and enhancement of HIV-specific immune responses. Therefore, we hypothesized that DCs may also have the capacity to present antigens from fusion competent nonreplicating virus i.e. AT-2 HIV-1[25], [44] to prime HIV-specific T cell responses. Here we show for the first time that AT-2 HIV-1 can serve as an efficient source of antigen for priming both CD4^+^ and CD8^+^ T cells and therefore function as a relevant source of antigen for priming in vivo.

The continuous presence of viral antigens gives rise to new memory T cells from the existing naïve population as long as the immune system remains functionally intact and/or the existing naïve T cells can respond to HIV-antigens [45] thus explaining the highly heterogeneous population of HIV-specific T cells that exist in HIV-infected individuals. The majority of the in vitro primed T cell responses that we observed were CD4^+^ T helper cells recognizing epitopes within Env (gp120^SU^, gp41^TM^, p24^CA^, p17^MA^), and Pol (RT and IN). The majority of the CD4^+^ T cell responses and all of the CD8^+^ T cell responses we observed in our priming targeted the same antigenic regions that had previously been documented in acutely and/or chronically HIV-infected subjects[19], [31], [36], [46], [47]. Individuals with acute or long term nonprogressive infection have a strong HIV-specific T cell ex vivo proliferative capacity, whereas this effector function seems to be absent in chronically infected individuals with high level of viremia [1], [2], [3]. The changes in functionality and phenotype of HIV-specific CD4^+^ T cells are a consequence of high levels of antigens [40]. The persistence of CD4^+^ T cells with defective proliferation suggests a dysfunction possibly due to impaired development of IL-2 producing central memory T cells [48], [49]. In contrast, the T cells activated in our in vitro priming are polyfunctional memory T cells, capable of secreting IL-2, IFN-γ, MIP-1β and TNF-α in response to stimulation. In addition, the HIV-specific T cells had the ability to recognize and proliferate in response to stimulation with both infectious and AT-2 HIV-1.

A number of the in vitro primed CD4^+^ T cell responses were towards peptides that had not previously been documented to elicit responses in the studies of infected individuals. When we investigated a cohort of acute/recent HIV-infected subjects, we detected responses to 4 of the 5 novel epitopes within env and pol that we found in our in vitro priming. Novel CD4^+^ T cell responses to the env gp41 peptides AVLSIVNRVRQGYSPLSF and LELDKWASLWNWFNITNW were seen in a couple of the donors used for priming. When we re-examined the CD4^+^ T cell responses of acute HIV-infected subjects for responses against these epitopes we were able to detect responses targeting the AVLSIVNRVRQGYSPLSF peptide. However, CD4^+^ T cell responses against this epitope waned as the infection progressed and were no longer detected at later time points. We have only observed a persisting response to this peptide in one subject that was chronically HIV-infected (data not shown).

On the other hand, CD4^+^ T cell responses to the peptide LELDKWASLWNWFNITNW env gp41_601-678_ so far remain undetected in acute and chronic HIV-1 infected subjects studied by our group and others (data not shown[19], [31], [36]). Interestingly, LELDKWASLWNWFNITNW corresponds to a highly conserved membrane proximal region of the gp41 and is only exposed during the fusion of the virus with the host membrane[50]. This epitope lies within a region identified as a CD4-epitope “hotspot” in mice immunized with HIV gp120[51], consistent with the idea that an otherwise immunodominant specificity is selectively eliminated in HIV-infected humans. Of note, this particular peptide also contains epitopes of two broadly neutralizing HIV-1 antibodies (2F5 and 4E10) that have been shown to be polyspecific autoantibodies reactive to phospolipid cardiolipin [52] Thus our inability to confirm responses to this particular peptide in HIV-infected subjects may be in part due to its autoimmune nature.

To further understand the skewing of priming to CD4^+^ T cells, we decided to examine CD4^+^ T cell responses in acute HIV-infected subjects. Our studies reveal that broad and strong CD4^+^ T cell responses are readily detected during AHI although some responses vanished within 1–2 months. Of note, CD4^+^ T cell responses targeting env are strong during this early stage and are detected as early as five days post-infection, whereas env responses are rarely detected in the later stages of infection[53], [54]. The mechanistic basis for the apparent preferential disappearance of HIV-specific CD4^+^ T cell responses of some specificities but not others, despite the ongoing presence of the relevant antigens, is unclear. In the case of HIV-specific CD8^+^ T cell responses there is also evidence of rapid disappearance of initially expanded cells and high avidity cells during AHI [55], [56]. This could be attributed to several mechanisms including mutations in the viral genome, inhibition of the presentation and processing of certain epitopes, and/or the inability of the T cells to adequately respond to survival signals. In the case for HIV-specific CD4^+^ T cells, an intriguing possibility is that these particular HIV-specific CD4^+^ T cells may be specifically targeted for deletion by the virus. In fact, we **(** **Figure 5B and 5C** **)** and others [34], [35] do provide convincing evidence for this particular theory. Of note, the activation of HIV-specific CD4^+^ T cell responses coincides with the presence of a widespread activation-induced CD4^+^ T cell apoptosis so that it is possible that these newly activated HIV-specific CD4^+^ T cell responses would be eliminated as a consequence of both specific and unspecific mechanisms. Alternatively, loss of HIV-1 specific CD4^+^ T cells may also be a consequence of chronic immune activation, e.g. due to bacterial translocation [57]. Regardless of the rationale, disappearance of these HIV-specific CD4^+^ and CD8^+^ T cell responses during AHI may ultimately contribute to the eventual inability of the immune system to control viral replication. In contrast, the persistence of HIV specific CD4^+^ T cells in untreated LTNPs highlights the importance of these cells. The helper activities of antigen-specific CD4^+^ T cells have been shown to mediate the control of many viral infections and are critical in maintaining HIV-1 suppression[1], [3], [58]. However there is individual heterogeneity in adaptive immune responses among LTNPs and consequently the correlates of immunity remain unknown. Therefore the key to deciphering this mystery may lie with the answer to why these HIV-specific CD4^+^ and CD8^+^ T cell responses disappear during acute HIV-1 infection [**59**].

In summary, our in vitro studies highlight the ability of DCs to efficiently prime naïve T cells and induce a broad repertoire of CD4^+^ T cells. Our findings provide important evidence showing that in a vaccine setting, AT-2 HIV-1 can not only restimulate memory T cell responses but is also capable of priming broad and polyfunctional de novo T cell responses. And given the recent failure of the Merck STEP Trial [60], the need to explore alternative vaccine platforms such as the therapeutic use of DC pulsed with AT2-HIV becomes critical in attaining the ultimate goal of an HIV vaccine.

## Materials and Methods

### Ethics Statement

This study was conducted according to the principles expressed in the Declaration of Helsinki. The study was approved by the Institutional Review Board of Massachusetts General Hospital and the Fenway Community Heath Care Center, Boston or via Center for HIV/AIDS Vaccine Immunology (CHAVI) clinical sites . All patients provided written informed consent for the collection of samples and subsequent analysis.

### Culture medium, cytokines, and reagents

Culture medium RPMI 1640 (Mediatech, Herndon, VA) or Yssel's T cell medium (Gemini Bio-Products, Sacramento, CA) was supplemented with 20ug/ml gentamicin (Gibco BRL, Gaithersburg, MD) and 1mM HEPES (Mediatech), and 1% human plasma or 5% PHS (Valley Biomedical, Winchester, VA). rhGM-CSF (Immunex, Seattle, WA) and rhIL-4 (R&D Systems, Minneapolis, MN) were obtained for the generation of DCs. rhIL-12, rhIL-6, and rhIL-7 (R&D Systems) and rhIL-2 (Chiron Corp, Emeryville, CA) were purchased for the priming cultures.

### Dendritic cells

Leukopacks were purchased from BRT Laboratories, Inc. (Baltimore, MD) Donors were tested prior to their initial donation and again at each donation for the presence antibodies to HBV, HCV, HTLV-I and II, HIV-1/HIV-2 and syphilis. PBMCs were separated by density gradient centrifugation on Ficoll-Hypaque (Amersham Pharmacia Biotech, Piscataway, NJ) DCs were grown from adherent PBMCs supplemented with 300U/ml IL-4 and 100IU/ml GM-CSF. After five days, immature DCs were collected, transferred to new plates, and induced to mature by adding a cocktail consisting of 150ng/ml IL-6, 5ng/ml TNF, and 5ng/ml IL-1β (R&D Systems) and 1 μg/ml PGE_2_ (Sigma).

### Viruses and infection of cells

Virions of HIV-1_MN_ (X4-tropic), clade B were produced by infection of CL.4/CEMX174 (T1) cells and purified by sucrose gradient ultracentrifugation as described previously[61]. Samples were titrated for the presence of infectious virus using AA2CL.1 cells and HIV-1 p24 antigen capture kits (AVP, NCI). Aldrithiol-2 (AT-2)-inactivated HIV-1 (AT-2 HIV) was prepared as described previously[62]. Used in this study include infectious HIV-1_MN_ (Lots 3807, 3966, and 3941: AVP, SAIC Frederick, Inc., NCI Frederick) and AT-2 HIV-1_MN_ (Lots 3808, 3965, 3937, and 3934). 1500ng/10^6^ p24 equivalents of HIV-1 was added to the cytokine-matured DCs on day six and incubated overnight (14–16 hours) at 37^°^C and after incubation the DCs were washed.

### Priming and Restimulation of T cells

Naïve CD4^+^ and CD8^+^ T cells were isolated by negative selection from frozen PBMCs, using magnetic beads (mb) to remove monocytes (CD14mb), B cells (CD19mb), NK cells (CD56mb), and memory T cells (CD45ROmb) (Miltenyi Biotec, Auburn, CA) and co-cultured with the HIV-pulsed MDDCs at a 1:10 or 1:15 ratio. Priming cultures were restimulated at day 7, 14, 21, and 28 with the addition of autologous HIV-pulsed MDDCs to the priming cultures.

### Measurement of expansion of antigen-specific CD8+ and CD4+ T cells after 7 days expansion by recall ELISPOT

Mature MDDCs pulsed with microvesicles, AT-2 or infectious HIV-1 were cultured with autologous bulk T cells at 1:10–1:30 ratios for 7 days. The expanded T cells were harvested and added to IFN-γ ELISPOT plates at 50,000 T cells/well and restimulated with DCs infected with vaccinia-vectors (MOI = 2) (V-ctr, V-env, V-gag, V-pol, V-nef) or pulsed with control, gp160, nef, p66, or p24 proteins (5 μg/ml; Protein Sciences Corporation, Meriden, CT). The ratio of DCs to T cells was 1∶10. The ELISPOT assays were carried out as described above. DCs infected with vaccinia-vectors were used to monitor CD8+ T cell responses[63] and HIV-protein pulsed MDDCs were used to monitor CD4+ T cell responses.

### Mapping of Epitopes

Mapping of epitopes was done using pools of overlapping peptides based on the consensus clade B sequence spanning the entire HIV proteome (Los Alamos immunology database). The peptides were mostly 18-mers and overlapped by 10 amino acids. To facilitate the screening of the peptides, peptide pool matrices were used in ELISPOT assays as described previously[19]. Reconfirmations of positive wells were done with single peptides.

### Assessment of polyfunctionality of primed CD4+ and CD8+ T cells

Analysis of the capacity of primed CD4+ and CD8+ T cells to secrete multiple cytokines/chemokines was done by initially stimulating them with MDDCs pulsed with 1500ng**/**10^6^ p24 equivalents HIV-1 for 6 hours with 10 μg/ml of the intracellular transport inhibitor brefeldin A (BFA; Sigma) before staining for the intracellular production of cytokines/chemokines.

### Antibodies and flow cytometry

MDDCs were stained with PE-conjugated antibodies to CD80, CD83, CD86, CD40, CD209, MHC class I and MHC class II (BD Pharmingen San Jose, CA). Primed T cells were stained with fluorochrome-conjugated antibodies to CD3, CD4, CD8, and IFN-γ (BD Pharmingen). For the assessment of cytokine/chemokine production, primed T cells were stained with fluorochrome-conjugated antibodies to IL-2, TNF, MIP-1β, and IFN-γ (BD Pharmingen). Flow cytometry analysis was performed on a FACSCalibur or LSRII (BD Biosciences). Data was analyzed using Cell Quest (BD Biosciences) or FlowJo (Tree Star, Inc, Ashland, OR) software.

### Subjects with acute or recent HIV infection

24 individuals with acute or early HIV-infection were enrolled in this study. Acute HIV-infection (n = 9) was defined by the presence of HIV-RNA in the plasma, a negative or weakly positive HIV-antibody by HIV-1/2 ELISA, and the detection of no more than three bands in an HIV Western blot (n = 3); recent HIV infection was defined by a positive ELISA and confirmed by detuned negative ELISA or confirmed previously negative ELISA. All but one of participants had symptoms compatible with the acute retroviral syndrome and the first blood sample available for immunological studies (baseline) was drawn at a median of 12 days (range 5–27) after onset of symptoms. Nine subjects were treated with HAART after this visit whereas three remained without antiretroviral therapy. Study participants were recruited either from the Massachusetts General Hospital and the Fenway Community Heath Care Center, Boston or via Center for HIV/AIDS Vaccine Immunology (CHAVI) clinical sites. All individuals gave written consent to participate, and the study was approved by the respective institutional review boards and conducted in accordance with the human experimentation guidelines.

## References

[1] Rosenberg ES, Billingsley JM, Caliendo AM, Boswell SL, Sax PE (1997). Vigorous HIV-1-specific CD4+ T cell responses associated with control of viremia.. Science.

[2] Rosenberg ES, Walker BD (1998). HIV type 1-specific helper T cells: a critical host defense.. AIDS Res Hum Retroviruses.

[3] Younes SA, Yassine-Diab B, Dumont AR, Boulassel MR, Grossman Z (2003). HIV-1 viremia prevents the establishment of interleukin 2-producing HIV-specific memory CD4+ T cells endowed with proliferative capacity.. J Exp Med.

[4] Zaunders JJ, Munier ML, Kaufmann DE, Ip S, Grey P (2005). Early proliferation of CCR5(+) CD38(+++) antigen-specific CD4(+) Th1 effector cells during primary HIV-1 infection.. Blood.

[5] Gauduin MC, Yu Y, Barabasz A, Carville A, Piatak M (2006). Induction of a virus-specific effector-memory CD4+ T cell response by attenuated SIV infection.. J Exp Med.

[6] MartIn-Fontecha A, Sebastiani S, Hopken UE, Uguccioni M, Lipp M (2003). Regulation of dendritic cell migration to the draining lymph node: impact on T lymphocyte traffic and priming.. J Exp Med.

[7] Macagno A, Napolitani G, Lanzavecchia A, Sallusto F (2007). Duration, combination and timing: the signal integration model of dendritic cell activation.. Trends Immunol.

[8] Saito H, Dubsky P, Dantin C, Finn OJ, Banchereau J (2006). Cross-priming of cyclin B1, MUC-1 and survivin-specific CD8+ T cells by dendritic cells loaded with killed allogeneic breast cancer cells.. Breast Cancer Res.

[9] Walsh SR, Bhardwaj N, Gandhil RT (2003). Dendritic cells and the promise of therapeutic vaccines for human immunodeficiency virus (HIV)-1.. Curr HIV Res.

[10] Lu W, Arraes LC, Ferreira WT, Andrieu JM (2004). Therapeutic dendritic-cell vaccine for chronic HIV-1 infection.. Nat Med.

[11] Huang XL, Fan Z, Zheng L, Borowski L, Li H (2003). Priming of human immunodeficiency virus type 1 (HIV-1)-specific CD8+ T cell responses by dendritic cells loaded with HIV-1 proteins.. J Infect Dis.

[12] Zarling AL, Johnson JG, Hoffman RW, Lee DR (1999). Induction of primary human CD8+ T lymphocyte responses in vitro using dendritic cells.. J Immunol.

[13] Gruber A, Kan-Mitchell J, Kuhen KL, Mukai T, Wong-Staal F (2000). Dendritic cells transduced by multiply deleted HIV-1 vectors exhibit normal phenotypes and functions and elicit an HIV-specific cytotoxic T-lymphocyte response in vitro.. Blood.

[14] Wilson CC, Olson WC, Tuting T, Rinaldo CR, Lotze MT (1999). HIV-1-specific CTL responses primed in vitro by blood-derived dendritic cells and Th1-biasing cytokines.. J Immunol.

[15] Bebenek K, Abbotts J, Roberts JD, Wilson SH, Kunkel TA (1989). Specificity and mechanism of error-prone replication by human immunodeficiency virus-1 reverse transcriptase.. J Biol Chem.

[16] Bebenek K, Roberts JD, Kunkel TA (1992). The effects of dNTP pool imbalances on frameshift fidelity during DNA replication.. J Biol Chem.

[17] Bebenek K, Thomas DC, Roberts JD, Eckstein F, Kunkel TA (1993). Effects of 3′-azido-3′-deoxythymidine metabolites on simian virus 40 origin-dependent replication and heteroduplex repair in HeLa cell extracts.. Mol Pharmacol.

[18] Thomas JA, Ott DE, Gorelick RJ (2007). Efficiency of human immunodeficiency virus type 1 postentry infection processes: evidence against disproportionate numbers of defective virions.. J Virol.

[19] Kaufmann DE, Bailey PM, Sidney J, Wagner B, Norris PJ (2004). Comprehensive analysis of human immunodeficiency virus type 1-specific CD4 responses reveals marked immunodominance of gag and nef and the presence of broadly recognized peptides.. J Virol.

[20] Frahm N, Korber BT, Adams CM, Szinger JJ, Draenert R (2004). Consistent cytotoxic-T-lymphocyte targeting of immunodominant regions in human immunodeficiency virus across multiple ethnicities.. J Virol.

[21] Larsson M (2004). HIV-1 and the hijacking of dendritic cells: a tug of war.. Springer Semin Immunopathol.

[22] Muthumani K, Hwang DS, Choo AY, Mayilvahanan S, Dayes NS (2005). HIV-1 Vpr inhibits the maturation and activation of macrophages and dendritic cells in vitro.. Int Immunol.

[23] Smed-Sorensen A, Lore K, Walther-Jallow L, Andersson J, Spetz AL (2004). HIV-1-infected dendritic cells up-regulate cell surface markers but fail to produce IL-12 p70 in response to CD40 ligand stimulation.. Blood.

[24] Granelli-Piperno A, Golebiowska A, Trumpfheller C, Siegal FP, Steinman RM (2004). HIV-1-infected monocyte-derived dendritic cells do not undergo maturation but can elicit IL-10 production and T cell regulation.. Proc Natl Acad Sci U S A.

[25] Larsson M, Fonteneau JF, Lirvall M, Haslett P, Lifson JD (2002). Activation of HIV-1 specific CD4 and CD8 T cells by human dendritic cells: roles for cross-presentation and non-infectious HIV-1 virus.. Aids.

[26] Sabado RL, Babcock E, Kavanagh DG, Tjomsland V, Walker BD (2007). Pathways utilized by dendritic cells for binding, uptake, processing and presentation of antigens derived from HIV-1.. Eur J Immunol.

[27] Larsson M, Fonteneau JF, Bhardwaj N (2003). Cross-presentation of cell-associated antigens by dendritic cells.. Curr Top Microbiol Immunol.

[28] McDonald D, Wu L, Bohks SM, KewalRamani VN, Unutmaz D (2003). Recruitment of HIV and its receptors to dendritic cell-T cell junctions.. Science.

[29] Day CL, Kaufmann DE, Kiepiela P, Brown JA, Moodley ES (2006). PD-1 expression on HIV-specific T cells is associated with T-cell exhaustion and disease progression.. Nature.

[30] Addo MM, Yu XG, Rathod A, Cohen D, Eldridge RL (2003). Comprehensive epitope analysis of human immunodeficiency virus type 1 (HIV-1)-specific T-cell responses directed against the entire expressed HIV-1 genome demonstrate broadly directed responses, but no correlation to viral load.. J Virol.

[31] Addo MM, Altfeld M, Rosenberg ES, Eldridge RL, Philips MN (2001). The HIV-1 regulatory proteins Tat and Rev are frequently targeted by cytotoxic T lymphocytes derived from HIV-1-infected individuals.. Proc Natl Acad Sci U S A.

[32] Yu XG, Lichterfeld M, Perkins B, Kalife E, Mui S (2005). High degree of inter-clade cross-reactivity of HIV-1-specific T cell responses at the single peptide level.. AIDS.

[33] Addo MM, Altfeld M, Rathod A, Yu M, Yu XG (2002). HIV-1 Vpu represents a minor target for cytotoxic T lymphocytes in HIV-1-infection.. Aids.

[34] Lore K, Smed-Sorensen A, Vasudevan J, Mascola JR, Koup RA (2005). Myeloid and plasmacytoid dendritic cells transfer HIV-1 preferentially to antigen-specific CD4+ T cells.. J Exp Med.

[35] Douek DC, Brenchley JM, Betts MR, Ambrozak DR, Hill BJ (2002). HIV preferentially infects HIV-specific CD4+ T cells.. Nature.

[36] Wilson CC, Palmer B, Southwood S, Sidney J, Higashimoto Y (2001). Identification and antigenicity of broadly cross-reactive and conserved human immunodeficiency virus type 1-derived helper T-lymphocyte epitopes.. J Virol.

[37] Karlsson H, Larsson P, Wold AE, Rudin A (2004). Pattern of cytokine responses to gram-positive and gram-negative commensal bacteria is profoundly changed when monocytes differentiate into dendritic cells.. Infect Immun.

[38] Cavrois M, Neidleman J, Kreisberg JF, Greene WC (2007). In vitro derived dendritic cells trans-infect CD4 T cells primarily with surface-bound HIV-1 virions.. PLoS Pathog.

[39] Zaunders JJ, Ip S, Munier ML, Kaufmann DE, Suzuki K (2006). Infection of CD127+ (interleukin-7 receptor+) CD4+ cells and overexpression of CTLA-4 are linked to loss of antigen-specific CD4 T cells during primary human immunodeficiency virus type 1 infection.. J Virol.

[40] Palmer BE, Blyveis N, Fontenot AP, Wilson CC (2005). Functional and phenotypic characterization of CD57+CD4+ T cells and their association with HIV-1-induced T cell dysfunction.. J Immunol.

[41] Kaufmann DE, Kavanagh DG, Pereyra F, Zaunders JJ, Mackey EW (2007). Upregulation of CTLA-4 by HIV-specific CD4+ T cells correlates with disease progression and defines a reversible immune dysfunction.. Nat Immunol.

[42] Lu W, Wu X, Lu Y, Guo W, Andrieu JM (2003). Therapeutic dendritic-cell vaccine for simian AIDS.. Nat Med.

[43] Frank I, Santos JJ, Mehlhop E, Villamide-Herrera L, Santisteban C (2003). Presentation of exogenous whole inactivated simian immunodeficiency virus by mature dendritic cells induces CD4+ and CD8+ T-cell responses.. J Acquir Immune Defic Syndr.

[44] Buseyne F, Le Gall S, Boccaccio C, Abastado JP, Lifson JD (2001). MHC-I-restricted presentation of HIV-1 virion antigens without viral replication.. Nat Med.

[45] Jabbari A, Harty JT (2006). Secondary memory CD8+ T cells are more protective but slower to acquire a central-memory phenotype.. J Exp Med.

[46] Berzofsky JA, Bensussan A, Cease KB, Bourge JF, Cheynier R (1988). Antigenic peptides recognized by T lymphocytes from AIDS viral envelope-immune humans.. Nature.

[47] Kent SJ, Greenberg PD, Hoffman MC, Akridge RE, McElrath MJ (1997). Antagonism of vaccine-induced HIV-1-specific CD4+ T cells by primary HIV-1 infection: potential mechanism of vaccine failure.. J Immunol.

[48] Palmer BE, Boritz E, Wilson CC (2004). Effects of sustained HIV-1 plasma viremia on HIV-1 Gag-specific CD4+ T cell maturation and function.. J Immunol.

[49] Okoye A, Meier-Schellersheim M, Brenchley JM, Hagen SI, Walker JM (2007). Progressive CD4+ central memory T cell decline results in CD4+ effector memory insufficiency and overt disease in chronic SIV infection.. J Exp Med.

[50] Burton DR, Desrosiers RC, Doms RW, Koff WC, Kwong PD (2004). HIV vaccine design and the neutralizing antibody problem.. Nat Immunol.

[51] Brown SA, Stambas J, Zhan X, Slobod KS, Coleclough C (2003). Clustering of Th cell epitopes on exposed regions of HIV envelope despite defects in antibody activity.. J Immunol.

[52] Haynes BF, Fleming J, St Clair EW, Katinger H, Stiegler G (2005). Cardiolipin polyspecific autoreactivity in two broadly neutralizing HIV-1 antibodies.. Science.

[53] Malhotra U, Holte S, Zhu T, Delpit E, Huntsberry C (2003). Early induction and maintenance of Env-specific T-helper cells following human immunodeficiency virus type 1 infection.. J Virol.

[54] Pitcher CJ, Quittner C, Peterson DM, Connors M, Koup RA (1999). HIV-1-specific CD4+ T cells are detectable in most individuals with active HIV-1 infection, but decline with prolonged viral suppression.. Nat Med.

[55] Pantaleo G, Soudeyns H, Demarest JF, Vaccarezza M, Graziosi C (1997). Evidence for rapid disappearance of initially expanded HIV-specific CD8+ T cell clones during primary HIV infection.. Proc Natl Acad Sci U S A.

[56] Lichterfeld M, Yu XG, Mui SK, Williams KL, Trocha A (2007). Selective depletion of high-avidity human immunodeficiency virus type 1 (HIV-1)-specific CD8+ T cells after early HIV-1 infection.. J Virol.

[57] Brenchley JM, Price DA, Schacker TW, Asher TE, Silvestri G (2006). Microbial translocation is a cause of systemic immune activation in chronic HIV infection.. Nat Med.

[58] Rosenberg ES, Altfeld M, Poon SH, Phillips MN, Wilkes BM (2000). Immune control of HIV-1 after early treatment of acute infection.. Nature.

[59] Pereyra F, Addo MM, Kaufmann DE, Liu Y, Miura T (2008). Genetic and immunologic heterogeneity among persons who control HIV infection in the absence of therapy.. J Infect Dis.

[60] Sekaly RP (2008). The failed HIV Merck vaccine study: a step back or a launching point for future vaccine development?. J Exp Med.

[61] Ott DE, Nigida SM, Henderson LE, Arthur LO (1995). The majority of cells are superinfected in a cloned cell line that produces high levels of human immunodeficiency virus type 1 strain MN.. J Virol.

[62] Rossio JL, Esser MT, Suryanarayana K, Schneider DK, Bess JW (1998). Inactivation of human immunodeficiency virus type 1 infectivity with preservation of conformational and functional integrity of virion surface proteins.. J Virol.

[63] Engelmayer J, Larsson M, Lee A, Lee M, Cox WI (2001). Mature dendritic cells infected with canarypox virus elicit strong anti-human immunodeficiency virus CD8+ and CD4+ T-cell responses from chronically infected individuals.. J Virol.

